# Inhibition of Return in Fear of Spiders: Discrepant Eye Movement and Reaction Time Data

**DOI:** 10.1155/2014/183924

**Published:** 2014-07-03

**Authors:** Elisa Berdica, Antje B. M. Gerdes, Andre Pittig, Georg W. Alpers

**Affiliations:** Department of Psychology, Clinical and Biological Psychology and Psychotherapy, School of Social Sciences, University of Mannheim, L13, 15-17, 68131 Mannheim, Germany

## Abstract

Inhibition of return (IOR) refers to a bias against returning the attention to a previously attended location. As a foraging facilitator it is thought to facilitate systematic visual search. With respect to neutral stimuli, this is generally thought to be adaptive, but when threatening stimuli appear in our environment, such a bias may be maladaptive. This experiment investigated the influence of phobia-related stimuli on the IOR effect using a discrimination task. A sample of 50 students (25 high, 25 low in spider fear) completed an IOR task including schematic representations of spiders or butterflies as targets. Eye movements were recorded and to assess discrimination among targets, participants indicated with button presses if targets were spiders or butterflies. Reaction time data did not reveal a significant IOR effect but a significant interaction of group and target; spider fearful participants were faster to respond to spider targets than to butterflies. Furthermore, eye-tracking data showed a robust IOR effect independent of stimulus category. These results offer a more comprehensive assessment of the motor and oculomotor factors involved in the IOR effect.

## 1. Introduction

We have a limited capacity to process all of the visual information that enters our visual field at any point in time. For this reason, selective attention to salient stimuli is necessary as it helps us decide where to move our eyes next [1]. In this regard, our attentional system enhances the processing of relevant information and diminishes the processing of less relevant information. This preferential detection can be visible in healthy individuals [2] but it is particularly enhanced in anxiety-prone individuals who are vigilant in detecting threat [3–7] and show disengagement deficits later on [7]. This quick detection of threat is evolutionary adaptive; however, when it interferes with everyday activities and generalizes to neutral stimuli, it can exacerbate the individual's anxious state [8].

When studying attentional biases and inhibitory processes, a relevant phenomenon is inhibition of return (IOR). This refers to an attentional bias against returning the attention to a previously attended location. It was first demonstrated by Posner and Cohen [9] in a spatial cueing paradigm. They presented participants a cue and subsequently a target which appeared in the cued or uncued location. Participants had to press a button when the target appeared. They found a facilitation effect for short stimulus-onset asynchronies (SOAs—the time between the presentation of the cue and the start of the presentation of the target) and an inhibitory aftereffect for longer SOAs (300–3000 ms). Facilitation refers to shorter reaction times to valid trials in comparison to invalid trials for short SOAs (0–300 ms SOA) while inhibition refers to longer reaction times to valid trials in comparison to invalid trials for longer SOAs (300–3000 ms). This inhibitory aftereffect suggests that more time is needed to redirect the attentional system to previously attended than to unattended locations. Its function is considered to be a foraging facilitator and is thought to help optimize visual search [10]. An increased likelihood to inspect new areas is adaptive when it comes to finding food or possible sources of threat. This mechanism suggests that search would not be efficient if we kept returning to locations that were inspected before.

Research on IOR is scant in clinical psychology but may be particularly relevant to anxiety disorders. This delayed response to previously attended locations may be less adaptive when individuals have to detect a threatening target. Inhibition of threatening information and facilitation of positive information would be a more plausible behavior. For this reason, recent research has started to examine the question whether threatening stimuli can actually interrupt this phenomenon when it is adaptive to pay attention to a fear-evoking cue and not just inhibit its processing. This would be in line with an evolutionary perspective. Until now, however, IOR has been shown to be very robust, nonflexible, even when emotional stimuli were used as cues [11–13]. In a study which employed a simple detection task, we were also unable to find a reduced IOR effect for spider cues and targets, in comparison to butterflies in a high spider fearful group [14]. On the other side, some studies seem to suggest that IOR is not completely immune and can be interrupted in some anxiety-related emotional states such as obsessive compulsive disorder [15], trait anxiety [16–18], and worry [19].

Previous work has mainly involved detection tasks; participants were instructed to press a button to localize the target. In their everyday life people are, however, faced with the need to discriminate and make judgments among a huge variety of stimuli. Therefore, the use of discrimination tasks may be more suitable when it comes to the processing of these emotional stimuli. Such discrimination tasks are used widely in the study of IOR and there is an ongoing debate concerning the time course of the IOR effect in discrimination. Lupiáñez and colleagues [20] argued that in a discrimination task the IOR effect appears later and disappears sooner in comparison to simple detection tasks. When combining discrimination and pictorial stimuli, we are aware of only one previous study which used biologically relevant stimuli in a discrimination task [11]. Taylor and Therrien based the discrimination on the identity of the target (discrimination among face and nonface targets). In the first two experiments they found a larger IOR effect for face targets in comparison to nonface targets when the target was made task relevant. In a third experiment the IOR effect for face targets emerged later than for nonface targets, suggesting that additional processing time may be needed when a task-relevant face target is presented. In their study eye movements were not prohibited.

While constraining the eye movements is common for IOR tasks, one could argue that this might not be representative for normal viewing conditions. Previous research has indeed demonstrated that the IOR effect has a close relationship to eye movements. The attentional and oculomotor components of the IOR effect were evaluated by Hunt and Kingstone [21]. They demonstrated that these components are often independent of one another offering a dissociation between the attentional and motor components of IOR. According to them, IOR reflects a bias against allocating covert attention to a previously cued location when the eyes remain stationary and a bias against executing a saccade to the cued location when the eyes are free to move to the target. However, until now, studies which combine eye-tracking and manual reaction times in an IOR task with fearful stimuli are missing; most of them only use neutral stimuli such as squares, circles, or dots.

Therefore, the present study investigates saccadic reaction times and manual reaction times in an IOR discrimination task. Eye movements were monitored while participants had to manually discriminate among emotional targets. This study further investigated whether the magnitude of IOR would be modulated by the emotional relevance of the targets that were used. Neutral stimuli were used as cues. In such tasks, the emotionality of the target might be more relevant since the response needs more time to develop. While the reflexive nature of the IOR might suggest that the mechanism underlying this effect should be insensitive to the emotionality of the stimulus that appears after the cue, we propose that the cue should not inhibit the processing of the target, when this target is threatening. For this reason, neutral cues and emotional targets were used.

It was expected that the spider fearful group would show a reduced IOR effect in comparison to the control group when spider targets appear, that is, no (or less) inhibition to validly cued targets. This reduction of the IOR effect is not expected to be visible for butterfly targets. It was also possible to investigate the way IOR affects later discrimination of the target; the same amount of IOR was expected for both short and long SOAs. It was further predicted that eye movements and manual reaction times go to the same direction.

## 2. Method

### 2.1. Participants

Sixty participants were recruited from the general population and from the student population of the University of Mannheim. Students received partial course credit and participants from the general population received information about spider phobia in exchange for their participation. Participants were selected according to their scores on the German version of the Fear of Spiders Questionnaire (FSQ, see [22]). Following Rinck et al. [22], participants with scores between 0 and 6 were assigned to the control group and participants with a score of 15 or higher to the fearful group. All participants had normal or corrected-to-normal vision. Exclusion criteria were serious medical conditions, substance abuse or dependence, and current use of psychotropic medication. All participants volunteered to participate and provided written informed consent prior to the experiment. The procedures were approved by the ethic committee of the University of Mannheim. In total, 60 participants completed the experiment. Data from seven participants were excluded due to technical failure during the eye-tracking recording. In addition, three participants were excluded because they did not follow the experimental instructions. Thus, the total sample for all further analyses consisted of 50 participants: 25 spider fearful and 25 control participants.

Table 1 shows demographic and questionnaire data for both groups. Statistical analysis supported that fear of spiders was significantly higher in fearful compared to control participants. The average level of fear of spiders for the spider fearful participants in the present study was comparable to the level of clinical samples with spider phobia in other studies [23].

No significant group differences were found for sex ratio, trait anxiety, and state anxiety at the beginning of the experiment but significant differences were apparent for spider fear and age. Age was included as a covariate in further analyses, but there was no significant effect of this factor on the general IOR effect. This analysis was not reported in the results to avoid redundant information.

### 2.2. Stimuli

**Figure 1:**
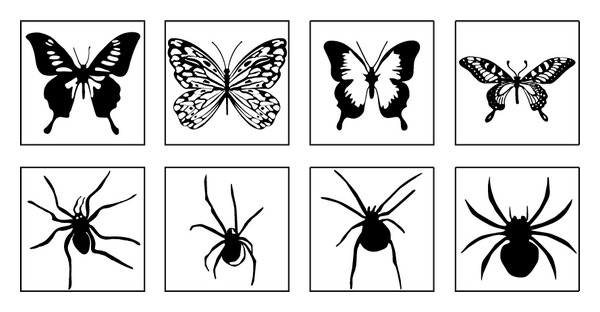
Example of the stimuli used in the experiment.

Black-and-white drawings of spiders and butterflies were chosen from the Internet and were enclosed into square frames. They were adjusted with Adobe Photoshop for their size and brightness and with ElectroMagnetic EncephaloGraphy Software (EMEGS) for their contrast [24]. At the end the stimuli consisted of 12 butterflies and 12 spiders, and a dot (see Figure 1). The picture size and orientation were identical to procedures used elsewhere [14]. Thus, the pictures were about 110 × 110 pixels in size (visual angle 3.3°); the frames were 146 × 146 pixels in size (visual angle 4.3°) and they were situated 197 pixels away from the fixation cross. Stimuli were presented with presentation software (Neurobehavioral Systems) on a 22 inch monitor with a resolution of 1024 by 768 pixels.

### 2.3. Eye Tracking

Eye monitoring was performed with an SMI RED250 eye-tracking device. It automatically tracked eye movements and compensated for head movements to ensure accurate and reliable results with a sampling rate of 250 Hz and tracking resolution of 0.03°. For the data analysis BeGaze eye-tracking analysis software was used. The areas of interest consisted of a square surrounding the frames where the stimuli appeared. They were about 146 × 146 pixels in size. The other area of interest consisted of a circle around the fixation cross. The saccades that were taken into consideration for further analyses were the eye movements from the fixation cross area to the target area of interest.

### 2.4. Procedure

After informed consent was obtained, participants completed a questionnaire battery. One questionnaire assessed sociodemographic data (age, sex, profession, handedness, smoking, and caffeine consumption) and whether participants had normal or corrected-to-normal vision. In order to control for individual levels of trait and state anxiety prior to the experimental paradigm, unspecific state and trait anxiety were assessed with the State-Trait Anxiety Inventory (STAI: [25]; German version: [26]). Fear of spiders was assessed with two self-report questionnaires; the Spider Phobia Questionnaire (SPQ: [27]; German version: [22]) and the Fear of Spiders Questionnaire (FSQ: [28]; German version: [22]). After completion of the questionnaire battery, participants were seated approximately 50 cm away from the monitor. After they read the instructions and completed the practice trials, they went through the 6-point calibration process, which involved fixating on a dot as it moved to different screen locations. Once the calibration was complete, the experiment (the IOR task) commenced.

**Figure 2:**
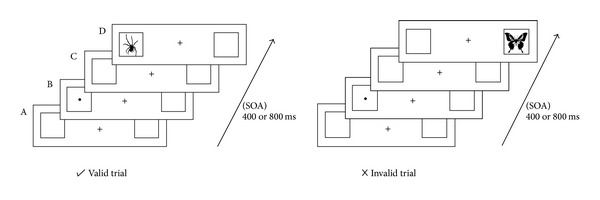
Sequence of events in a valid and invalid trial: (A) two empty frames appeared on the left and right of the fixation cross; (B) cue was presented for 200 ms in one of the frames; (C) two empty frames appeared again for 400 ms or 800 ms; (D) target stimulus was presented in the cued or uncued location.

An example of a valid and invalid trial is shown in Figure 2. Each trial started with a presentation of the two empty frames on the left and right of the fixation cross for 500 ms (A). Afterwards a cue (always a dot) appeared in one of the frames for 200 ms (B), which was followed by another screen with empty frames and the fixation cross (C). The* stimulus onset asynchrony* (SOA) was either 400 ms or 800 ms long. After the SOA interval, a butterfly or a spider was presented as target stimulus in one of the frames until the participants responded or for a maximum duration of 2 s (D). They were instructed to make a saccade in the direction of the target as fast as possible and then indicate which of the two pictures was presented with a button press. To discriminate between both types of target stimuli (spiders and butterflies), they were instructed to press the “arrow up” or the “arrow down” key. The button-picture assignment was counterbalanced (i.e., 50% of the participants had to press “arrow up” for the spider and “arrow down” for the butterfly, whereas this assignment was reversed for the other 50%). The “arrow up” and “arrow down” were chosen as response keys to prevent interference of keys on responses towards the stimuli presented to the left or right of the fixation cross.

The validity of the cue depended on the position relative to the target stimulus in each trial. Following the typical IOR task, in valid trials, cue and target stimulus were presented at the same location whereas in invalid trials, they were presented at different locations.

Fifty practice trials were used to familiarize participants with the task and to ensure that they understood and followed instructions. The subsequent experimental trials varied in terms of three experimental factors: (1) target stimulus type (spider versus butterfly); (2) validity of the cue (valid versus invalid); and (3) the stimulus onset asynchrony (SOA) interval (400 ms versus 800 ms). For each of the eight conditions (target stimulus ∗ validity ∗ SOA), 42 experimental trials were presented (8 different conditions all counterbalanced), which resulted in a total of 312 experimental trials. The order of these experimental trials was pseudorandomized and the different conditions (validity, target stimulus type, and SOA) were combined equally often.

At the end of the task, participants were again asked to fill in the state version of the STAI in order to assess changes in state anxiety after the task. In addition, they rated each spider and butterfly stimulus using the 9-point* Self-Assessment Manikin* (SAM) rating system [29]. The SAM is a picture-based rating system to directly measure the valence (from “1” pleasant to “9” unpleasant) and emotional arousal (from “1” not at all aroused to “9” extremely aroused) associated with the individual reaction to a different stimuli. The entire experiment lasted 45 minutes.

### 2.5. Statistical Analyses

Two dependent variables were measured. First,* entry times* were calculated using eye-tracking data. Entry time was defined as the first saccade after target onset issued from the fixation cross to the correct target stimulus. Errors in the eye movement task were considered trials where participants' eyes moved to the direction of the cue first, or when the saccade was missing completely. In the beginning of the experiment participants were instructed to never move their eyes toward the cue. There were, though, a few error trials per participant. This was easy to detect as the eyes are not in the fixation cross area of interest in the moment when the saccade starts. So these error trials were excluded from further analyses. The practice trials served as a way to get used to the procedure so that during the experiment there were only a few errors occurring.

For outlier correction, all entry times below 158 ms and above 398 ms (two standard deviations smaller and bigger than the general mean score) were excluded from further analysis. Second,* reaction times* were calculated as time between the presentation of the target stimulus and the participant's discrimination response. For outlier correction, reaction times below 439 ms and above 838 ms (again, two standard deviations from the general mean score) were excluded from further analysis. To make sure that the outlier correction is accurate, a further analysis was conducted. The cutoffs were computed based on the SD separately for each anxiety group. The same results were obtained. Only the RT changed with about 10 ms. In addition, in this case it was necessary to add and subtract only one SD from the general mean, while in the general outlier correction two SDs were added and subtracted, as for the errors in the eye movement task.

Both entry and reaction time data were analyzed separately with a 2 × 2 × 2 × 2 analysis of variance (ANOVA) with cue validity (valid versus invalid), target stimulus (spider versus butterfly), and SOA interval (400 ms versus 800 ms) as within-subject factor and fear of spiders (fearful versus nonfearful) as between-subject group factor. According to Lupiáñez et al. [20] the IOR effect is different for short and long SOAs in discrimination; therefore separate analysis for the 400 ms SOA and 800 ms SOA was further conducted in our experiment.

In addition, valence and arousal ratings were analyzed as a manipulation check in order to verify that spider fearful participants rated the spider pictures as less pleasant and more arousing. To this end, valence and arousal ratings were analyzed with a 2 × 2 ANOVA with target stimulus (spider versus butterfly) as within-subject factor and fear of spiders (fearful versus nonfearful) as a between-subject group factor, respectively.

## 3. Results

### 3.1. Manipulation Check

The pictures we presented as targets induced the expected emotional responses. For valence ratings, the repeated measures ANOVA yielded a significant main effect of picture category, *F*(1,45) = 140, *P* < 0.001,  *η*_*p*_^2^ = 0.75, and a significant interaction of picture category and fear of spiders, *F*(1,45) = 22.8, *P* < 0.001, *η*_*p*_^2^ = 0.34. Spider fearful participants rated the pictures of spiders as more unpleasant (spiders: *M* = 2.59, SD = 1.27, and butterflies: *M* = 7.13, SD = 0.90) in comparison to the control participants (spiders: *M* = 4.35, SD = 0.81, and butterflies: M = 6.28, SD = 0.90): *t*(45) = 5.67, *P* < 0.001 for spider valence rating and *t*(45) = 2.55, *P* = 0.014 for butterfly valence rating.

For arousal ratings, the repeated measures ANOVA again revealed a significant main effect of picture category, *F*(1,45) = 54, *P* < 0.001, *η*_*p*_^2^ = 0.54, and a significant interaction of picture category and fear of spiders, *F*(1,45) = 24.3, *P* < 0.001, *η*_*p*_^2^ = 0.35. Spider fearful participants rated spider pictures as significantly more arousing (*M* = 6.21, SD = 2.4) in comparison to the control participants (*M* = 3.35, SD = 1.8), *t*(45) = 4.53, *P* < 0.001, but no statistically significant difference was detected for ratings of butterflies pictures (fearful participants: *M* = 2.53, SD = 1.41; control participants: *M* = 2.63, SD = 1.57, *t*(45) = −0.227, *P* = 0.821). Thus, the stimulus material used in this experiment was rated as expected among fearful and control participants.

### 3.2. Eye-Tracking Data: Entry Times

**Figure 3:**
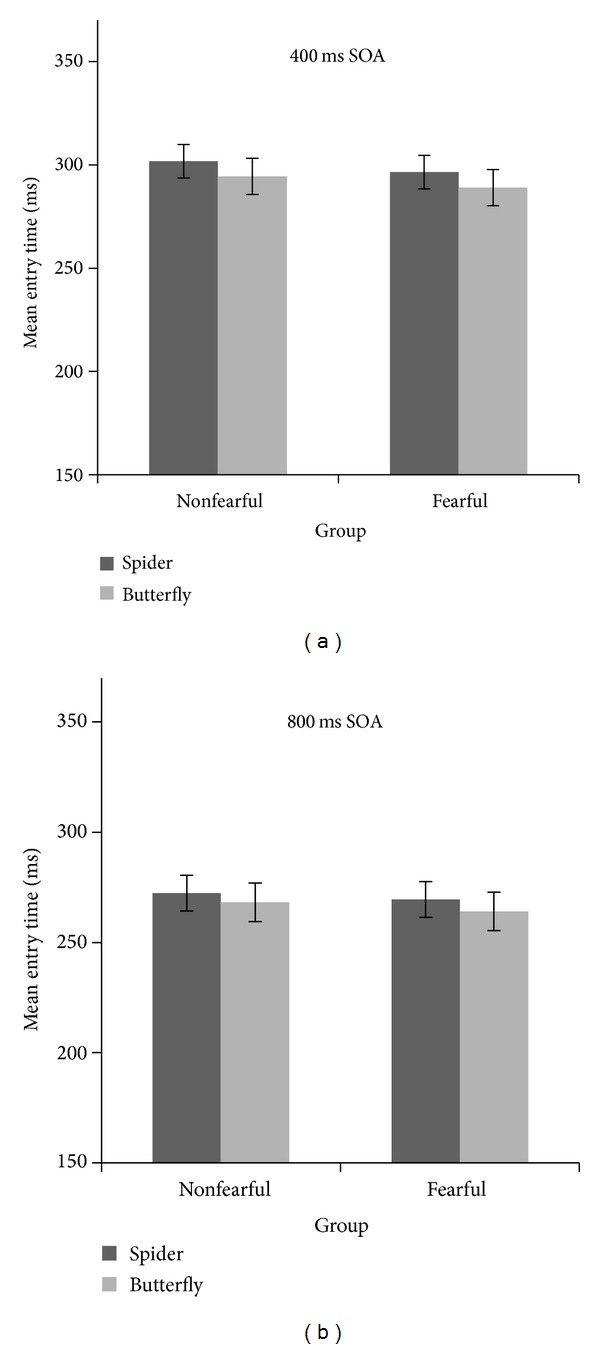
Eye-tracking data—mean entry time on target area in milliseconds for the control group and spider fearful group for the 400 ms SOA (a) separately for spider and butterfly targets and for the 800 ms SOA (b). Bars represent the standard error of the mean.

The factors that were used for the analyses include cue validity (valid versus invalid), target stimulus (spider versus butterfly), SOA interval (400 ms versus 800 ms), and fear of spiders (fearful versus nonfearful). Eye-tracking data revealed an IOR effect which was not modulated by emotional target content. The ANOVA with mean entry time as a dependent variable revealed a main effect of cue validity, *F*(1,48) = 47.2; *P* < 0.001, *η*_*p*_^2^ = 0.49 (*M* = 292,18 for valid and *M* = 271,8 for invalid trials); SOA: *F*(1,48) = 75.9; *P* < 0.001, *η*_*p*_^2^ = 0.61 (*M* = 295.45 for the 400 ms SOA and *M* = 268.54 for the 800 ms SOA); target stimulus, *F*(1,48) = 8.38; *P* = 0.006, *η*_*p*_^2^ = 0.15, and a significant interaction of Cue Validity × SOA: *F*(1,48) = 6.68; *P* = 0.013, *η*_*p*_^2^ = 0.12 (suggesting that the IOR effect is different for the two SOAs used in the experiment: stronger for short SOAs and weaker for long SOAs). The Cue Validity × Fear of Spiders interaction was not significant: *F*(1,48) = 1.79; *P* = 0.18, *η*_*p*_^2^ = 0.04. Likewise the Cue Validity × Target Stimulus interaction was not significant: *F*(1,48) = 0.10; *P* = 0.75, *η*_*p*_^2^ = 0.002 suggesting that the IOR effect is not modulated by the emotionality of the target (Figure 3).

To further explore the effect of the different SOAs used in the experiment, separate analyses were conducted for the 400 ms SOA and for the 800 ms SOA. For the 400 ms SOA, there was a main effect of cue validity, *F*(1,48) = 43.2; *P* < 0.001, *η*_*p*_^2^ = 0.47, and a main effect of the target stimulus *F*(1,48) = 9.61; *P* = 0.003, *η*_*p*_^2^ = 0.16. While for the 800 ms SOA condition, the analysis revealed only a main effect of cue validity, *F*(1,48) = 23.4; *P* < 0.001, *η*_*p*_^2^ = 0.32, meaning that there was an IOR effect for the entire group independently of stimulus type or fear of spiders and there was no interaction of Cue Validity × Target Stimulus × Fear of Spiders *F*(1,48) = 0.11; *P* = 0.74, *η*_*p*_^2^ = 0.00 (Figure 3).

### 3.3. Motor Response Data: Reaction Times

Manual responses did not show an IOR effect—there was no main effect of cue validity *F*(1,48) = 0.35, *P* = 0.55, *η*_*p*_^2^ = 0.007, but a main effect of the target stimulus, *F*(1,48) = 18.6, *P* < 0.001, *η*_*p*_^2^ = 0.28 (*M* = 604.51 for spider targets and *M* = 618.51 for butterfly targets): in general both groups were faster to press the button for spiders than for butterflies. More specifically, there was a significant interaction of Target Stimulus × Fear of Spiders, *F*(1,48) = 4.96, *P* = 0.03, *η*_*p*_^2^ = 0.09; that is, spider fearful participants are faster to press the button for spider targets than butterfly targets, in comparison to the control group. The interaction of Cue Validity × Target Stimulus was not significant, *F*(1,48) = 0.12, *P* = 0.72, *η*_*p*_^2^ < 0.001.

**Figure 4:**
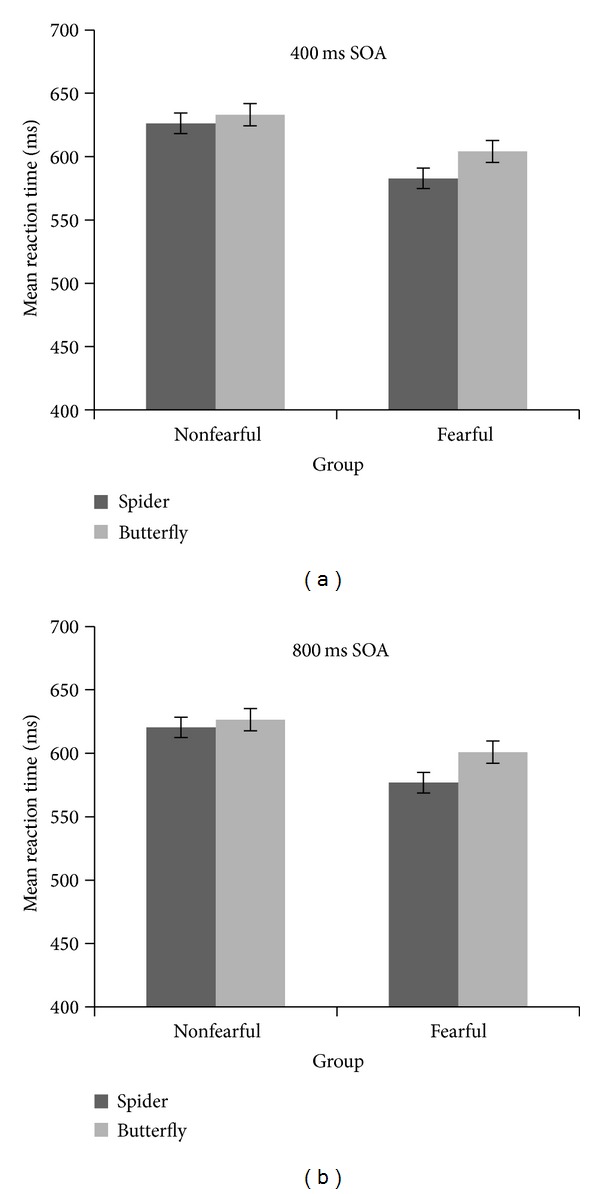
Mean reaction time in milliseconds for the control group and spider fearful group for the 400 ms SOA (a) separately for spider and butterfly targets and for the 800 ms SOA (b). Bars represent the standard error of the mean.

A further exploratory analysis for the two different SOAs revealed that this interaction was valid only for the 800 ms SOA, *F*(1,48) = 6.9, *P* = 0.01, *η*_*p*_^2^ = 0.12 (Figure 4(b)) but not for the 400 ms SOA, *F*(1,48) = 2.2, *P* = 0.14, *η*_*p*_^2^ = 0.04 (Figure 4(a)).

In sum, the analysis of the motor responses (the button press) did not show any IOR effect.

## 4. Discussion

Inhibition of return is thought to facilitate foraging behavior. Although the phenomenon is generally found to be very stable, it is plausible that it can be affected by emotional content of the stimuli which are presented. In the present study, eye-tracking was used to investigate the IOR effect with phobia relevant stimuli as targets. There was no evidence for the influence of fear on the IOR effect. However, separate analyses of the eye-tracking data and manual reaction times showed different patterns of the IOR effect. Eye-tracking data revealed a strong IOR effect independent of diagnostic group and independent of the target stimulus. This effect was mainly present in the 400 ms SOA condition while for the 800 ms SOA condition, this effect was relatively weak. Considering the two SOAs separately indicates that timing matters in discrimination; a stronger IOR effect is visible for shorter SOAs and less of an IOR effect for longer SOAs.

There were some other unexpected findings; we found that participants were generally faster to move their eyes toward the butterflies compared to spiders. We cannot conclusively argue that this is a result of the stimulus properties as they were controlled for contrast, color, and size. An explanation for this result, however, may be the shape of the butterflies which could capture the visual attention more easily. We are not aware of a comparable effect in the literature.

In contrast, the IOR effect was not found in the manual responses. Participants were generally faster to press the button for spiders than for butterflies but this effect was more pronounced in spider fearful participants. This may be interpreted as a sign of vigilance toward threatening stimuli but is in conflict to the eye-tracking data, where greater vigilance toward butterflies was found. Previous work recommends an enhanced hypervigilance toward all kinds of stimuli in spider fearful individuals [30], consistent with what we found in the manual reaction times. The hypervigilance of threat hypothesis would predict higher vigilance of spiders as evidenced by faster saccades toward spiders [31–35]. However, we found that participants detect butterflies faster with their eyes but are able to react more quickly toward spiders. The first response (turning the eyes in the direction of the target) likely did not depend on a semantic analysis, because the pictures which appeared in the periphery at first, low-level features may have driven the initial response. However, once the target was fixated the manual response was more likely to be influenced by specific features. Taking into account the fact that spider fear is spider specific [36] an explanation for this result might be the fact that spiders are more unambiguous than butterflies and they are processed preferentially in our brain when they are attended to [37]. Moreover, faster manual responses to spider targets might result from preparation for motor action in response to potential threat [38]. Thus, these discrepant results for saccadic and manual response times could depict the fact that not all aspects of attentional engagement, disengagement, and behavioral response to a task will be reflected in our multilevel assessments. In particular, readiness to respond to threatening stimuli may be different in the optical system (eye-tracking) and the motor system (manual response). As we conclude also from the present study, with an emotional stimulus response execution is speeded up. Future research should aim at dismantling these different components of the IOR effect.

Although there is a general discussion about the time course of the IOR effect during discrimination, the present results suggest that IOR is detectable for short and long SOAs. This runs contrary to Lupiáñez et al.'s observation that this effect is only observed for longer SOAs [20]. One could argue that this is not a typical discrimination procedure as participants responded to the targets first by moving the eyes toward them, whereas fixation was maintained in Lupiáñez work. Perhaps our results would be similar to other studies that suggest that IOR is not visible in discrimination [39] had we instructed participants not to move their eyes; however this would also have reduced the ecological validity of our design.

Our results are particularly interesting because we make use of both eye movement measures and reaction time measures. In line with previous studies, our finding suggested that eye movement latencies show IOR [40]. However, the eye movement differences did not result in an IOR effect in reaction time data. Still, in routine searches, people are expected to make eye movements toward targets and these eye movements are usually followed by motor responses. That is, action typically follows detection—when spider fearful individuals encounter a spider, the threat is first detected and then some action is taken. For this reason we chose not to constrain eye movements in our study. Previous IOR research has generally limited the procedures to only saccadic eye movements or manual reaction time responses, but this does not seem to accurately reflect behaviors that occur in natural settings. As Klein et al. [41] argue, attention plays a crucial role in the execution of eye movements and one of the goals of the present study was indeed to further examine whether IOR is reflected in the saccade latencies and/or in the motor responses. Such discrepancies have been observed by other groups who use similar assessments [42]. Using a change detection paradigm, they recorded eye movements and manual response times and also found discrepant findings—participant's eye movements and manual responses went into different directions.

The button press discrimination in the present study was used as a control measure to ensure that participants fully paid attention to the target stimulus. Discrimination of emotional and neutral stimuli using saccadic and manual responses was investigated previously also by others who showed that eye movements, in contrast to manual responses, require little information to distinguish emotional faces [43]. This interpretation is also in line with our findings. In the present study, all saccadic reaction times were quick in comparison to manual reaction times as well.

## 5. Conclusions

Taken together, our findings help to further extend the motor and oculomotor explanation of the IOR effect and provide greater support for the lack of a strong influence of emotional stimuli on this attentional bias. It seems to be a stable phenomenon and not easily interrupted by the emotional valence of the stimuli, even when potential threat is presented. This is not in line with the evolutionary perspective, which suggests that quick detection of threatening stimuli has survival value and the attentional capture in this case is automatic, but of course this is not to say that such mechanisms may appear under most circumstances.

This study combines findings from research on anxiety, inhibition of return, and attention with findings from eye movement research. We recommend that further research should focus on the oculomotor aspect of IOR and on the specific conditions under which this effect is weakened.

## Figures and Tables

**Table 1:** Demographics and questionnaire means and standard deviations for both fearful and control participants.

Measure	Control participants		Fearful participants			
	M	SD	M	SD	*t*(48)	*P*
Age	24.60	5.29	31.04	13.19	2.26	<0.001
FAS	2.80	3.12	52.32	25.94	9.47	<0.001
SPF	5.72	2.83	15.32	5.61	7.63	0.001
STAI-T	39.32	9.49	38.40	8.50	−0.36	0.624
STAI-S Before	36.24	7.82	37.56	9.60	0.53	0.817
STAI-S After	24.60	5.29	31.04	13.19	2.66	0.481

Note. Definitions of questionnaire abbreviations used in the table can be found in the procedure section.
